# Case of Rupture of the Aorta, near Its Arch

**Published:** 1827-07

**Authors:** Thomas Rose

**Affiliations:** Surgeon to St. George's Hospital.


RUPTURE OF THE AORTA, WITHOUT ANEURISM.

*Case of Rupture of the Aorta, near its Arch.* Communicated by Thomas Rose, Esq. Surgeon to St. George's Hospital.

*Park-place ; April 16th,* 1827.

Dear Sir,—I beg leave to transmit to you the account of an extensive rupture of the aorta, near its arch, which lately came under my observation. There was no aneurism, but the coats of the artery, from the commencement of the vessel at the semilunar valves to its bifurcation, were very easily torn; and the internal coat exhibited the thickened steatomatous appearance which might naturally have been expected to lead to the formation of that disease. It seems, indeed, not improbable that this was actually forming during the few hours of suffering which preceded the patient's death, by the internal and middle coats of the vessel having given way, and by the dilatation of the cellular coat. The latter, however, and the adjacent parts, were unable to resist the impetus of the circulation; and, after injecting to a great extent the cellular tissue on the outsides of the pleura and pericardium, the blood forced its way through these membranes, and the hemorrhage speedily proved fatal.

I remain, dear Sir, faithfully yours,

THOMAS ROSE.

*To Dr. Macleod.*

A gentleman, fifty-two years of age, of a stout and large frame of body, and of rather a full habit, who had at different times suffered a good deal of anxiety of mind, was attacked very suddenly, on the evening of the 16th of February, with a sharp pain commencing in his chin, and passing rapidly down his neck, in the course of the great vessels, to his chest and back. He was engaged in earnest conversation with a friend at the moment when it seized him, but it was so severe as to oblige him almost immediately to retire to bed. He passed a restless night, the pain continuing unabated during it, as it did during the succeeding day.

At six o'clock on the afternoon of that day, (the 17th,) I saw him by desire of his son, a very attentive pupil of St. George's Hospital, and of Mr. Miles, formerly house surgeon of that hospital, who was at the time on a visit in his house. I found him lying on his back, with his arms extended by his side. He seemed afraid to turn himself in bed or to move. His pulse was soft, and easily compressed, about ninety-six or one hundred; his tongue was moist, and had a brown tinge over its centre; his countenance expressed much anxiety. He could fully distend his lungs, but this, like every other exertion, was followed by an increase of pain. He described the pain as very severe, and said it radiated about his kidneys and back, and occasionally passed across his chest. His bowels had acted once, but not very freely. He had taken a dose of infusion of Rhubarb and Senna in the early part of the day; but Mr. Miles informed me that the pulse at first appeared so languid, that he had judged it advisable to give one or two doses of Ammonia in Camphor mixture, before he had ventured to administer the aperient draught.

There was nothing in the previous history of his complaints to throw any light on the nature of this attack. He had been affected with rheumatic pains about a fortnight before, chiefly attacking his loins and sacrum, but occasionally extending along every part of his spine; and had at that time been cupped on the loins to the extent of a few ounces. He had often complained of pains in his shoulders, and in the folds of his arms; but these had not been much noticed, although his friends observed that his appearance had not been of late by any means so healthy as formerly.

I was unable to determine to what cause to refer his sufferings, and directed a common saline aperient mixture to be given in repeated doses, until his bowels should be freely acted upon; to which, at the suggestion of Mr. Miles, a little Vinum Colchici was added. I returned a little before eleven. He had vomited the first dose of the medicine, but thought himself much relieved by it, and his pulse and tongue had become more natural. He passed a very quiet night, without the assistance of an opiate which had been directed to be had in readiness, in case it should be required; and, when I saw him on the morning of the 18th, he stated that he felt himself much better in every respect, and that the pain which he experienced was then comparatively trifling.

At nine in the evening of the 18th, I was again called to see him. He had continued in bed during the day, taking merely a little broth occasionally, which he ate with appetite, and he had been easy and cheerful. Once or twice he had complained to his son of a noise, which he thought proceeded from some people knocking on the partition between his room and the next; and he insisted on orders being sent to stop it, though nothing of the kind could be heard by others. It appeared that he had made the same complaints on the preceding day. About eight o'clock in the evening, he became restless, got up repeatedly from a disposition to tenesmus, which was not relieved by an injection of starch and opium; and, when I saw him at nine, he was laid across his bed, in a state of extreme depression, having a feeble, very irregular, and fluttering pulse; a cold perspiration on his skin, and coldness of his extremities. Upon being got into bed, and on some strong port-wine negus being administered, the circulation soon became more active, and his pulse became more firm. I sent his son to request Dr. Hewett to visit him, who came immediately. Before his arrival, however, the alarming symptoms of depression had a good deal subsided, and, though still languid and feeble, he was able to give a clear account of his feelings, and to describe the pain which he had experienced.

Dr. Hewett ordered a few grains of Calomel and Ipecacuan, with extract of Poppies, to be given immediately, and to be repeated during the night; and that he should take some Castor-oil in the morning. He directed also a mixture of Ammonia and Camphor mixture, to be given occasionally, in case the faintness should recur.

At four in the morning, I was again summoned to see him, but before I reached his house he was dead. It appeared that he continued quiet and easy until near that hour, when the same disposition to tenesmus returned, obliging him to get out of bed two or three times. After being up for a few minutes, the faintness returned, and he shortly afterwards expired.

I obtained permission to examine the body, which was done on the 21st, in the presence of Dr. Hewett and Mr. Miles, and the following were the morbid appearances:—

On opening into the pericardium, about three ounces of serum, mixed with blood, were found in its cavity. The basis of the heart, and the commencements of the great vessels, presented a completely ecchymosed appearance, from blood having been forced into the cellular membrane between them and that part of the pericardium which is reflected over them. At one small spot that membrane had given way, from the effusion between it and the muscular fibres of the heart; and through this the blood had passed which was found in the cavity of the pericardium.

The semilunar valves at the root of the aorta were partially ossified, at that part of their margin which is attached to the sides of the vessel. Beyond these, the whole internal membrane of the artery, as far as its bifurcation, presented an irregular thickened appearance, from numerous, whitish, flattened tubercles, of a steatomatous character, being every where deposited in it. It could be lacerated with the greatest ease.

At the arch of the aorta, there was a rent of not less than two inches in length on its concave side, beginning in the ascending portion, and terminating immediately opposite the origin of the left carotid and subclavian. There was no appearance of ulceration at the edges of this rent. An enormous effusion of blood had taken place every where about the vessel. A tumor consisting of recent coagulum, of twice the bulk of a lemon, surrounded that part of the vessel in which the rent had taken place. The posterior mediastinum was completely filled with blood, which was also injected extensively between the lungs and the pleura on each side, more especially the left; and, in short, every where into the cells of the cellular membrane in the thorax. The pleura on each side had been ruptured by the pressure of the blood; more than two quarts of coagulated blood and serum being found in the cavity of the left pleura, and about half as much in that of the right.

It is difficult to determine at what precise period the rupture of the vessel took place. It appears to me that the internal coats gave way on the first attack,—that is, on the evening of the 16th; the cellular coat restraining for a time the effusion of blood, and commencing the formation of aneurism, which would probably have been the result had the rent been less extensive, or the impetus of the circulation more moderate. The blood thrown out round the vessel would account for the languid state of the patient's pulse, and for the distressing pain which he experienced in his chest and back. During the efforts which he made to evacuate his bowels, the quantity of effused blood was no doubt materially increased, gradually filling the cellular membrane in the thorax, and producing the alarming faintness on the evening of the 17th; and the rupture of the pleura on each side was probably the immediate forerunner of death.

*Memoranda of two Cases of Rupture of the Aorta, without Aneurism.*

RUPTURE of the aorta without aneurism appears to be a rare occurrence. The following case, which recently fell under the notice of Mr. ARNOTT, shows that laceration may take place in a healthy vessel, as a consequence of violent concussion of the body, from the shock of falling from a height.

A bricklayer, falling from a scaffold forty feet high, severely injured the head and chest. He lived an hour after the accident, as I was informed by the gentleman who saw him professionally, in a state of insensibility, with a cold skin and feeble pulse.

On examination of the body, which was that of a strong middle-aged man, besides extensive fracture of the cranium and fracture of the lower jaw, the following circumstances were observed:—Fracture of four true ribs on the right side of the chest, and three of the left, without displacement. Slight extravasation of blood into the cellular substance of the anterior mediastinum, at its upper part; that of the posterior so loaded as to present the appearance of one mass of soft coagulum. The aorta at the concave part of its arch, immediately beyond the origin of the left subclavian, but on the opposite side of the vessel, and where it turns to become descendens, presented a complete rent in its coats, the laceration forming a very oblong aperture, about three-quarters of an inch in length, and one-eighth across at its widest part. The line of laceration was straight, but oblique as regarded the axis of the vessel. The coats of the artery at this point, as well as elsewhere, offered no evidence of previous disease, but appeared perfectly healthy. No injury of the mediastinum could be discovered, and the aperture of the aorta did not seem in any way connected with the fractured extremities of the ribs. The blood poured out from the rent in the vessel, having been confined to the cellular substance of the posterior mediastinum, and this being a space of bounded extent, seems to be the reason why its occurrence did not prove immediately fatal.

*New Burlington-street; May* 10*th*, 1827.

We find also, in an early Number of this Journal, the case of Thomas Jones, boatswain of his Majesty's ship Thames, who, early in the morning of the 24th October, 1806, was heard to jump out of his hammock, and to groan. On a light being brought, he was found completely dead. His body was covered with perspiration. This man, from the account of Mr. JAMES, the surgeon, had been doing his duty with his accustomed alacrity the day before, and appeared in perfect health when he went to bed: indeed, he was never known to complain during the time he was on board the frigate, which was nearly a twelvemonth.

On opening the cavity of the thorax, and slitting open the pericardium, it was found distended with blood, part of which was in a coagulated state. On examining from whence this blood proceeded, the aorta was found ruptured about an inch from the semilunar valves: this rupture was about half an inch in length. Small particles of calcareous matter were found deposited at the bottom of the mitral valves, and the corpora sesamoidea aurantii seemed to be composed in toto of this substance. The coats of the aorta were perfectly sound, as not the least deposition, either of ossific or calcareous matter, could be detected about the place where the rupture was situated. The abdominal viscera were perfectly healthy.*

